# Household Composition of Older U.S. Adults With Cognitive Difficulties by Race and Ethnicity

**DOI:** 10.1093/geroni/igaf122.581

**Published:** 2025-12-31

**Authors:** Jennifer Kwok

**Affiliations:** University of Illinois Chicago, Chicago, Illinois, United States

## Abstract

Older adults with cognitive difficulties may receive informal support and care from family members in their households. This study investigated how the household composition of community-dwelling older adults with cognitive difficulties varies by sociodemographic characteristics, focusing on race and ethnicity. I used the U.S. Census Bureau’s American Community Survey Public Use Microdata Sample from 2008-2021. The 7.0% of community-dwelling adults aged 65 and older who are reported to have serious difficulty concentrating, remembering, or making decisions because of a physical, mental, or emotional condition were classified as having cognitive difficulties. I used logistic regression models that incorporated the survey design, individual characteristics (age, sex, education, income), and control variables (years) to understand associations between household composition and race and ethnicity in this population. Black / African American individuals had a significantly lower average predicted probability of living with spouses (31.4%; 95% confidence interval (CI): 16.7%-46.1%) than White (44.6%; CI:29.9%-59.2%), Asian / Pacific Islander (44.4%; CI:36.0%-52.9%), and Hispanic/Latino (43.4%; CI:38.5%-48.3%) individuals. Asian / Pacific Islander individuals had the highest average predicted probability of living with adult children (48.5%; CI:46.2%-50.7%), followed by Hispanic/Latino (41.3%; CI:36.6%-46.0%), Black / African American (33.5%; CI:32.0%-34.9%), and White (22.5%; CI:18.9%-26.2%) individuals. Results are similar in subpopulations of individuals who also have independent living difficulties and excluding 2020-2021 data. These findings suggest that policies that aim to help older adults with cognitive difficulties and their caregivers should consider that diverse U.S. populations likely vary in their access to and reliance on family members for informal support and care.

